# *Delirium* in cancer patients admitted to the intensive care unit: a retrospective study

**DOI:** 10.5935/0103-507X.20190064

**Published:** 2019

**Authors:** Bárbara Rocha Gouveia, Rafael Tavares Jomar, Tania Cristina de Oliveira Valente

**Affiliations:** 1 Instituto Nacional de Câncer José Alencar Gomes da Silva - Rio de Janeiro (RJ), Brasil.; 2 Universidade Federal do Estado do Rio de Janeiro - Rio de Janeiro (RJ), Brasil.

**Keywords:** Delirium/epidemiology, Neoplasms/complications, Respiration, artificial, Drug-related side effects and adverse reactions, Intensive care units

## Abstract

**Objective:**

To describe the occurrence of *delirium* in cancer patients admitted to the intensive care unit according to clinical and demographic characteristics.

**Methods:**

A retrospective study was conducted with 135 adults admitted to the intensive care unit of a public cancer hospital in the city of Rio de Janeiro, Brazil, between January and March 2016. Fisher's exact test and the linear association test were used to identify statistically significant associations between the occurrence of *delirium* and categorical and ordinal variables, respectively, considering a p-value < 0.05.

**Results:**

The overall occurrence of *delirium* was 39.3%. Delirium was more frequent among individuals aged 60 years or older and those who required extensive assistance or were bedbound, were admitted to the intensive care unit for clinical reasons, were using sedative drugs, were undergoing chemotherapy, and those who remained 8 or more days in the intensive care unit. Considering only patients on mechanical ventilation, the overall occurrence of *delirium* was 64.6%, and only a length of stay in the intensive care unit ≥ 8 days showed a statistically significant association with *delirium*.

**Conclusion:**

The occurrence of *delirium* in critically ill cancer patients is high. When only those on mechanical ventilation are considered, the occurrence of *delirium* is even greater.

## INTRODUCTION

*Delirium* is a disturbance of attention or awareness accompanied by a change in baseline cognition, which manifests as a reduced ability to direct, focus, sustain and shift attention because the individual is easily distracted by irrelevant stimuli. It occurs for a short period, usually lasting from hours to a few days, and has a tendency to fluctuate, worsening at dusk and at night, when external stimuli decrease. There is evidence that *delirium* is a physiological consequence of underlying medical conditions, substance intoxication or withdrawal, drug use, exposure to toxins, or a combination of these factors.^(1)^

*Delirium* in intensive care units (ICU) has an incidence ranging from 5% to 92%^(2)^ and is associated with longer hospital stays and increased mortality.^(3)^ In patients with cancer, the incidence of *delirium* is also high; it can reach 80% in more advanced stages of the disease and is related to worse pain control and decreased survival.^(4,5)^

A prospective study conducted at the ICU of the Brazilian National Cancer Institute in 2011 found a high prevalence of acute cerebral dysfunction (*delirium* and coma) in critically ill cancer patients on mechanical ventilation (95%) that surpassed that reported in the literature for groups of critically ill patients without cancer. The same study stated that when *delirium* is diagnosed and treated early, the quality of life improves, and the length of hospital stay and the number of readmissions decreases.^(6)^

Considering the lack of studies on *delirium* in cancer patients, the aim of this study was to describe the occurrence of *delirium* in patients admitted to an oncology ICU in terms of clinical and demographic characteristics.

## METHODS

**Figure 1:**
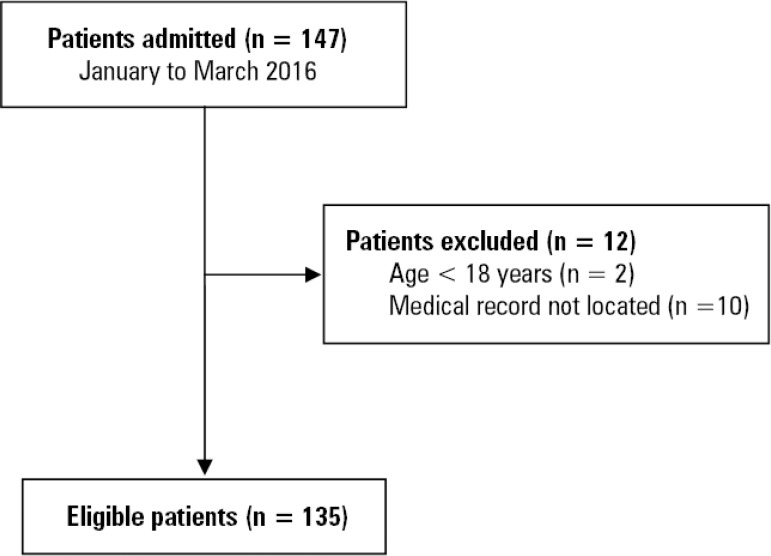
Flowchart of the retrospective cohort of cancer patients in the intensive care unit.

This retrospective study was based on the records of medical records of patients admitted to the ICU of a cancer hospital located in the city of Rio de Janeiro (RJ), Brazil. The intentional sample consisted of 135 patients aged ≥ 18 years who were diagnosed with cancer and admitted to the ICU between January and March 2016; the sample comprised 97.1% of the patients with cancer admitted to the ICU during that time period (Figure 1).

The following information was collected from the medical records by a trained ICU nurse: sex (male or female); age (in years); performance status according to the Performance Status of the Eastern Cooperative Oncology Group^(7)^ (independent, little assistance needed or considerable assistance needed/bedbound); type of admission (clinical or surgical); reasons for admission (postoperative monitoring, sepsis, respiratory failure, shock (except septic), neurological, renal/metabolic, cardiovascular, digestive, or other); use of vasoactive drugs (yes or no); use of sedative drugs (yes or no); receiving chemotherapy (yes or no); length of ICU stay (in days; arbitrarily categorized as ≤ 7 days and ≥ 8 days for analysis); receiving mechanical ventilation (yes or no); anatomical site of the primary tumor; distant metastasis (yes or no); and occurrence of *delirium* (yes or no), according to the Confusion Assessment Method for the Intensive Care Unit (CAM-ICU),^(8)^ which was applied for patients with scores higher than -4 (-3 to +4) on the Richmond Agitation and Sedation Scale (RASS),^(9)^ which, in turn, was applied daily (every 12 hours) in the studied ICU.

Using the Statistical Package for Social Sciences software, version 21.0, Fischer's exact test and the test of linear association were used to identify statistically significant associations between the occurrence of *delirium* and categorical variables (sex, age, type of admission, use of sedative drugs, chemotherapy and mechanical ventilation use) and ordinal variables (performance status and reason for admission), respectively, considering a p value < 0.05.

The Shapiro-Wilk normality test indicated that the duration of *delirium* (in days) was not normally distributed and violated the assumptions required for the use of parametric tests. Thus, to compare the duration of *delirium* in patients who remained in the ICU for up to 7 days with that of patients who remained in the ICU for 8 days or more, the Mann-Whitney U test, which is the nonparametric alternative to Student's t-test, was used.

The study was approved by the Research Ethics Committees of the *Universidade Federal do Estado do Rio de Janeiro* and the *Instituto Nacional de Câncer José Alencar Gomes da Silva* according to the National Health Council resolutions 466/2012 and 510/2016.

## RESULTS

The average length of stay in the ICU was 10.4 (± 12.1) days. The average duration of *delirium* was 2.1 (± 3.7) days, and its overall occurrence rate was 39.3% (n = 53). Solid tumors were diagnosed in 116 (85.9%) patients and included brain tumors (n = 25), colorectal tumors (n = 19) and oral cavity/pharyngeal tumors (n = 17). Nineteen (14.1%) patients were diagnosed with blood cancer, including multiple myeloma (n = 6), acute leukemia (n = 5), Hodgkin's lymphoma (n = 3), non-Hodgkin's lymphoma (n = 2), and chronic leukemia (n = 2). Distant metastasis was diagnosed in 37 (27.4%) patients. Regarding clinical status, 82 patients (60.7%) received vasoactive drugs, and 81 (60.0%) received sedative drugs during the ICU stay. Among the patients who experienced *delirium*, the main drugs used were dexmedetomidine (n = 44), fentanyl (n = 43) and midazolam (n = 31).

Table 1 shows the occurrence of *delirium* according to the patients' clinical and demographic characteristics. *Delirium* was more common among people aged 60 years or older and those who required considerable assistance/were bedbound, were admitted to the ICU due to clinical reasons, used sedative drugs, were receiving chemotherapy, or remained in the ICU for 8 or more days.

**Table 1:** Occurrence of *delirium* according to clinical and demographic characteristics (n = 135)

Variables	Delirium		p value
	No	Yes	
Sex			0.528
Male	45 (58.4)	32 (41.6)	
Female	37 (63.8)	21 (36.2)	
Age range (years)			0.049
18 - 59	42 (70.0)	18 (30.0)	
≥ 60	40 (53.3)	35 (46.7)	
Performance status			0.001
Independent	7 (77.8)	2 (22.2)	
Little assistance needed	46 (75.4)	15 (24.6)	
Considerable assistance needed/bedbound	28 (43.8)	36 (56.3)	
Type of admission			0.001
Clinical	23 (43.4)	30 (56.6)	
Surgical	59 (72.0)	23 (28.0)	
Reason for admission			0.045
Postoperative monitoring	56 (73.7)	20 (26.3)	
Sepsis	14 (46.7)	16 (53.3)	
Respiratory failure	02 (40.0)	03 (60.0)	
Shock (except septic)	02 (50.0)	02 (50.0)	
Neurological	02 (66.7)	01 (33.3)	
Renal/metabolic	01 (20.0)	04 (80.0)	
Cardiovascular	03 (37.5)	05 (62.5)	
Digestive	-	01 (100.0)	
Other	02 (66.7)	01 (33.3)	
Use of sedative drugs			< 0.001
No	50 (92.6)	04 (7.4)	
Yes	32 (39.5)	49 (60.5)	
Receiving chemotherapy			0.033
No	80 (63.0)	47 (37.0)	
Yes	02 (25.0)	06 (75.0)	
Days of ICU stay			< 0.001
≤ 7	67 (83.8)	13 (16.3)	
≥ 8	15 (27.3)	40 (72.7)	

ICU - intensive care unit. The results are expressed as n (%).

Considering only the patients on mechanical ventilation (n = 65), the overall occurrence of *delirium* was 64.6%. Table 2 shows the occurrence of *delirium* among patients receiving mechanical ventilation according to clinical and demographic characteristics. Among these variables, only a length of ICU stay ≥ 8 days showed a significant correlation.

**Table 2:** Occurrence of *delirium* among patients on mechanical ventilation according to clinical and demographic characteristics (N = 65)

Variables	Delirium		p value
	No	Yes	
Sex			0.244
Male	16 (41.0)	23 (59.0)	
Female	07 (26.9)	19 (73.1)	
Age range (years)			0.966
18 - 59	07 (35.0)	13 (65.0)	
≥ 60	16 (35.6)	29 (64.4)	
Performance status			0.482
Independent	02 (66.7)	01 (33.3)	
Little assistance needed	05 (33.3)	10 (66.7)	
Considerable assistance needed/bedbound	15 (32.6)	31 (67.4)	
Type of admission			0.652
Clinical	15 (37.5)	25 (62.5)	
Surgical	08 (32.0)	17 (68.0)	
Use of sedative drugs			0.456
No	-	01 (100.0)	
Yes	23 (35.9)	41 (64.1)	
Days of ICU stay			< 0.001
≤ 7	13 (76.5)	04 (23.5)	
≥ 8	10 (20.8)	38 (79.2)	

ICU - intensive care unit. The results are expressed as n (%).

## DISCUSSION

The occurrence of *delirium* among critical patients without cancer ranges from 9% to 80%^(10-14)^. Thus, the occurrence rate among the patients in the present study is within the parameters expected for critically ill patients without cancer.

In agreement with this study, *delirium* occurs in 10% to 34% of patients during the postoperative period for general surgery and specialties other than oncology.^(15,16)^ It is noteworthy that *delirium* is often associated with a significant increase in morbidity and mortality^(15,16)^ and, therefore, deserves special attention from the health team to avoid more severe outcomes. In noncritical hospital settings, *delirium* usually lasts approximately 1 week, although some symptoms usually persist even after discharge.^(17)^

A comparison among age groups found a significantly higher rate of *delirium* among participants older than 60 years. In this study, 21.4% of the sample was aged over 60 years; this agrees with the results of previous studies that show that the most patients most vulnerable to developing *delirium* are those older than 60 years, and occurrence rates can range between 10% and 30% in this population.^(18)^

A study that evaluated 1,515 terminal cancer patients found that *delirium* occurred in more than 43% of the sample.^(19)^ In the present study, the occurrence of *delirium* in patients who progressed to palliative care after an ICU stay was 7.5% (data not shown). In Japan, a cohort study was conducted with 180 patients in ICU at 24 medical centers to assess the incidence of *delirium* and its effects on 28-day mortality in critically ill patients on mechanical ventilation. In that study, 64% of the patients developed *delirium*; however, *delirium* was not associated with higher mortality as the ICU mortality rate was 8%.^(15)^

A descriptive study that analyzed the nursing team's records of perceived signs and symptoms of *delirium* included a 52.3% proportion of males;^(20)^ that proportion was close to that of our study, which was 41.6%. Although sex did not show a statistically significant difference in the occurrence of *delirium* in the present study (p = 0.528), nurses consider males more vulnerable than females to the development of *delirium*; this perception may be related to the beliefs and stereotypes of these professionals given that an important part of their interventions comprises measures to control the behavior of male patients, thus justifying immobilization measures.^(20)^

As expected, sedation was used by most of the patients in the sample that met clinical criteria for the diagnosis of *delirium*; the most frequently used drugs were dexmedetomidine (n = 44), fentanyl (n = 43) and midazolam (n = 31). McNicoll et al. reported that midazolam increases the likelihood of transitioning to *delirium*.^(21)^ Salluh et al. compared the use of dexmedetomidine with the use of lorazepam for reducing the duration of *delirium* and observed that sedation with dexmedetomidine resulted in more *delirium*- or coma-free days.^(22)^

Patients who were admitted to the ICU for up to 7 days had a mean *delirium* duration of 0.4 days (± 0.9), which was significantly lower (p < 0.001) than that of patients admitted for 8 days or more (mean = 4.6; ± 4.8). A prospective study conducted at the ICU of the Brazilian National Cancer Institute found that the mean length of ICU stay among patients on mechanical ventilation with *delirium*/coma was 14 days, while that of patients without *delirium* was 13 days; the differences was not statistically significant (p = 0.94).^(6)^ The main difference between the results of the aforementioned study^(6)^ and the present study is that the previous study included only patients on mechanical ventilation, which likely hinders the rapid reversal of *delirium*, as was observed among the patients with shorter ICU stays in this study.

In an observational study, Muller et al. reported that 61.8% of patients used mechanical ventilation.^(23)^ In our population, the rate of mechanical ventilation was 48.1%, and the rate of *delirium* was 64.6%, corroborating studies in which the frequency of *delirium* ranged from 60% to 80%.^(6,24,25)^

It is worth noting that this study requires interpretation in light of its limitations and strengths. Limitations include the fact we did not exclude patients with dementia when forming the cohort and the impossibility of constructing a multivariate regression model that could identify characteristics associated with the occurrence of *delirium* due to the small number of study participants. As strengths, we highlight the daily recording of the CAM-ICU^(8)^ and RASS^(9)^ scale results in the medical records, which contributed to the formation of a cohort without a loss of patients due to missing information regarding the occurrence of *delirium* (outcome) in the studied scenario.

## CONCLUSION

In critically ill cancer patients, the occurrence rate of *delirium* is high; when only patients on mechanical ventilation are considered, the rate of *delirium* occurrence is even greater.
